# A low fractional excretion of Phosphate/Fgf23 ratio is associated with severe abdominal Aortic calcification in stage 3 and 4 kidney disease patients

**DOI:** 10.1186/1471-2369-14-221

**Published:** 2013-10-12

**Authors:** Lourdes Craver, Adriana Dusso, Montserrat Martinez-Alonso, Felipe Sarro, José M Valdivielso, Elvira Fernández

**Affiliations:** 1 Nephrology Service and Unit for the Detection and Treatment of Atherothrombotic diseases (UDETMA), Hospital Universitari Arnau de Vilanova, Av Rovira Roure, 25198 Lleida, Spain; 2 Statistics Department, IRBLLEIDA, Av. Rovira Roure 80, Lleida, Spain; 3 Experimental Nephrology Laboratory, IRBLLEIDA, Av. Rovira Roure 80, 25198 Lleida, Spain

**Keywords:** Vascular calcification, FGF23, Fractional excretion of phosphate, Atherosclerosis, Soluble klotho

## Abstract

**Background:**

Vascular calcification (VC) contributes to high mortality rates in chronic kidney disease (CKD). High serum phosphate and FGF23 levels and impaired phosphaturic response to FGF23 may affect VC. Therefore, their relative contribution to abdominal aortic calcification (AAC) was examined in patients CKD stages 3–4.

**Methods:**

Potential risk factors for AAC, measured by the Kauppila Index (KI), were studied in 178 patients.

**Results:**

In multivariate linear analysis, AAC associated positively with age, male gender, CKD-stage, presence of carotid plaques (CP) and also with FGF23, but negatively with fractional excretion of phosphate (FEP). Intriguingly, FEP increased with similar slopes with elevations in PTH, with reductions in GFR, and also with elevations in FGF23 but the latter only in patients with none (KI = 0) or mild (KI = 1-5) AAC. Lack of a FEP-FGF23 correlation in patients with severe AAC (KI > 5) suggested a role for an impaired phosphaturic response to FGF23 but not to PTH in AAC. Logistic and zero-inflated analysis confirmed the independent association of age, CKD stage, male gender and CP with AAC, and also identified a threshold FEP/FGF23 ratio of 1/3.9, below which the chances for a patient of presenting severe AAC increased by 3-fold. Accordingly, KI remained unchanged as FEP/FGF23 ratios decreased from 1/1 to 1/3.9 but markedly increased in parallel with further reductions in FEP/FGF23 < 1/3.9.

**Conclusions:**

In CKD 3–4, an impaired phosphaturic response to FGF23 with FEP/FGF23 < 1/3.9 associates with severe AAC independently of age, gender or CP.

## Background

Cardiovascular disease is the main cause of mortality in chronic kidney disease (CKD) patients [1-4] and vascular calcification (VC) is a critical contributor to the progression of vascular lesions [5-7]. For decades, therapy has been directed to correct abnormalities in phosphate, calcium, and vitamin D metabolism, all of which cause elevations in parathyroid hormone (PTH), and predispose to VC [8,9]. Currently, the role of the FGF23 in the development of VC in the course of CKD is the focus of intense research, as serum FGF23 increases earlier than either phosphate or PTH and appears to mark a prolonged positive phosphate balance. Furthermore, in advanced CKD stages, as in the dialysis population, extremely high FGF23 levels associate with VC regardless of serum phosphate [10-12]. However, at earlier stages, conflicting reports exist as to whether high FGF23 but not serum phosphate [13], or high phosphate but not FGF23, independently associate with VC [14]. In support for the latter, neutralization of FGF23 in a rat model of CKD improves associated hyperparathyroidism but increases VC and mortality [15]. However, in animals with normal renal function, increases in FGF23 may protect from soft tissue calcifications through phosphaturic and PTH suppressive actions, and also through a potent inhibition of renal calcitriol production, which in turn limits intestinal calcium and phosphate absorption [16,17]. Furthermore, ex-vivo studies in human arteries and Vascular Smooth Muscle Cells (VSMC) have demonstrated anti-calcifying rather than pro-calcific actions of FGF23, provided there is sufficient arterial klotho for FGF23 actions [18]. Undoubtedly, the contradictory evidence on the role of high FGF23 on VC in CKD may result from the inability to discriminate between adaptive increases in serum FGF23 that translate into an adequate renal phosphaturic response from further elevations in FGF23 resulting from the failure of the damaged kidney to handle the phosphate load. Indeed, Dominguez et al. [19] have recently shown that in patients with cardiovascular disease but without CKD, the lower is the phosphaturic response of the kidney the higher is the association between serum FGF23 levels and adverse cardiovascular events. The high impact of VC on adverse cardiovascular events and mortality risk [20] led us to assess the influence of an abnormal renal handling of the phosphate/FGF23 axis on abdominal aortic calcification (AAC) in CKD patients stages 3 and 4.

High serum FGF23 also induces cardiovascular disease through mechanisms unrelated to abnormal phosphate homeostasis [21]. Because high FGF23 correlates directly with total body atherosclerosis [22], it is possible that high FGF23 enhancement of atheromatous disease progression may also contribute to VC. Indeed, in hemodialysis patients, atheromatous disease is also associated with arterial intima calcification [23]. Therefore, this work was designed to evaluate the relative impact of high serum FGF23 or of an impaired phosphaturic response to FGF23 on the severity of AAC in CKD patients stages 3 and 4.

## Methods

### Study design

Cross-sectional study that enrolled 205 patients CKD stages 3 and 4, according to K/DOQI guidelines [24], from the Division of Nephrology and the UDETMA Unit at the Hospital Universitario Arnau de Vilanova (HUAV) in Lleida, Spain. Final sample size was 178, as 27 patients were excluded due to history of primary hyperparathyroidism, neoplasia, parathyroidectomy, renal transplantation, and osteoporosis treated with biphosphonates or calcitonin, or treated with steroids (n = 8); lack of carotid ultrasound evaluating atheromatous lesions (n = 18), and the one black patient among Caucasians. Protocols were approved by the committee for human studies at the HUAV. Informed consent was obtained from all participants.

Data collected include: age, gender, CKD etiology, presence of diabetes, hypertension, pulse pressure, smoking status and prior history of cardiovascular disease. Estimated glomerular filtration rates (eGFR) were calculated using the Modification of Diet in Renal Disease (MDRD) equation [25]. At the time of initiation of recruitment (January 2008), neither calcium-free phosphate binder nor paricalcitol were available. Therefore, treatment with calcium-containing phosphate binders (for serum phosphate > 4.5 mg/dl) and/or calcitriol (for serum PTH > 20 pmol/l in CKD stage 3, or >25 pmol/l in stage 4), was either avoided or minimized in patients with prior history of vascular or soft tissue calcification.

### Laboratory data

Fasting venous blood samples and a 24 hour urine collection were obtained between 8–9 AM, to minimize daily circadian variations in serum phosphate levels. Routine tests included lipid profile, serum levels of glucose, albumin, C reactive protein (CRP), creatinine, phosphate, calcium, CaxP product, bone-specific alkaline phosphatase.

Selected parameters of mineral metabolism included: Serum levels of intact PTH (iPTH; by chemiluminescence immunoassay (Cobast®, Roche Diagnostics GmbH), 25-hydroxyvitamin D (25(OH)D) and 1,25-dihydroxyvitamin (calcitriol) by radioimmunoassay (Biosource®) and radioreceptor assay Gamma-B dihydroxyvitamin D, IDS Hybritec®, respectively. Vitamin D supplementation (400 IU/day) in vitamin D deficient patients eliminated seasonal differences in serum 25(OH)D in patients recruited during the winter (68.8%). Urinary calcium, sodium, and phosphate excretion in 24 h and their respective fractional excretions (FE) were measured. FEP = [Urinary P (mg/dl) × Serum Creatinine (mg/dl)] / [Serum P (mg/dl) × Urinary creatinine (mg/dl)] × 100. Protein intake was estimated using a standard formula, as previously described [26].

Serum FGF23 and soluble α-Klotho were measured with Elisa kits for human C-Term FGF23 (Immutopics, Inc., San Clemente, CA) and for soluble α-Klotho (Immuno-Biological Laboratories Co., Ltd., Japan), following manufacturer’s protocols with intra- and inter-assay coefficient of variation of 5%.

### Measurements of abdominal aortic calcification and carotid atheromatous disease

AAC measured by the Kauppila index (KI) [27] was obtained from lateral lumbar X rays, and evaluated independently by two highly experienced examiners. The inter-observer coefficient of variation was below 2%.

Carotid ultrasound (MicroMaxx, SonoSite with the linear transducer HFL38/13-6 MHz) measured the presence of carotid plaques (CP) and carotid intima-media thickness (IMT; semi-automated, FDA-approved software, Sono Calc IMT®), as previously described [28].

### Statistical analysis

Data are presented as mean ± SD for quantitative variables, and as percentage of patients for descriptive, qualitative variables. A logarithmic transformation of serum FGF23 allowed achieving a distribution close to normal. KI tertiles defined patients as: Non-calcified (KI = 0), moderately calcified: KI values ≥1 up to 5; and severely calcified: KI higher than 5. The statistical significance of the differences among the 3 KI-based subgroups was measured with Kruskal-Wallis for quantitative variables and chi-square tests for qualitative variables (or Fisher test for expected frequencies below 5). Differences in KI, FEP and FGF23 among CKD 3 and 4 were assessed using the Kruskal-Wallis test. The Pearson correlation coefficient measured the linear correlation between KI values as the dependent variable with each of every other quantitative variable in bivariate analysis.

A scatterplot, with linear and nonparametric regression lines assessed the relationship between FEP/FGF23 in logarithmic scale (base 2) with the Kauppila index. The non parametric regression to delineate mean fitting was implemented as a local polynomial surface regression with a smoothing degree of 0.96. For dispersion from the mean fit, we used an estimate of the square root of the variance function, with separate smoothing of the squares of the positive and negative residuals from the mean fit. This analysis identified the ratio FEP/FGF23 = 1/3.9 as a critical cutoff point further used in multivariate analysis. Multivariate linear regression and logistic regression analyses identified the variables that contributed significantly to either explain the variability in KI as a continuous variable, or to discriminate patients without AAC (KI = 0) or with severe AAC (KI > 5), respectively. The significance of interaction effects of KI tertiles on the relationship of FGF23, PTH or eGFR with FEP was assessed using Likelihood Ratio (LR) tests. The area under the ROC curve (AUC) assessed the discrimination capability of the logistic model and the Hosmer-Lemeshow goodness-of-fit tests measured model calibration. A zero-inflated regression model was used to simultaneously fit the high frequency of KI = 0 in our patient population with actual KI scores. Due to overdispersion, the Negative Binomial distribution was chosen over the Poisson distribution to fit KI scores. The explanatory variables chosen minimized residual deviance when comparing hierarchical models according to LR tests. Data were analyzed using the software: “Statistics for the Social Sciences” SPSS 11.0, and/or the free software R. For all tests, a p < 0.05 was considered statistically significant.

## Results

Table 1 shows socio-demographical and clinical data in all patients and in the three subgroups defined by tertiles of KI. KI subgroups differed in gender, etiology of CKD, smoking status, previous history of cardiovascular disease (CHF specifically), presence of carotid plaques, severity of the atheromatous disease, and in the number of patients receiving treatment with anti-thrombotic and diuretic drugs at the time AAC was measured. KI subgroups also differed in age, pulse pressure, IMT, serum FGF23 levels, and CaxP product, but had similar serum creatinine, phosphate, calcium, PTH, estimated GFR, and daily protein/phosphate intake.

**Table 1:** Characteristics of the overall sample and subgroups defined by KI values

	**Overall sample**	**Subgroups defined by Kauppila Index**			
**Characteristics**	**(n = 178)**	**KI = 0 (n = 57)**	**KI 1–5 (n = 68)**	**KI > 5 (n = 53)**	**P value**
**Female**	37.6 (67)	52.6 (30)	29.4 (20)	32.1 (17)	0.017
**Age**	69.1 ± 11.6	61 ± 14	71 ± 8	75 ± 6	0.001
**BMI (Kg/m2)**	28.5 ± 5.2	28.2 ± 6.1	29.2 ± 4.5	27.9 ± 4.9	0.342
**Systolic BP (mmHg)**	138.9 ± 21.2	134 ± 22	142 ± 19	140 ± 22	0.118
**Diastolic BP (mmHg)**	72.6 ± 10.9	74 ± 10	73 ± 11	70 ± 12	0.123
**Pulse pressure (mmHg)**	66.3 ± 19.9	60 ± 21	69 ± 19	70 ± 19	0.011
***CKD Stage***					0.169
**CKD 3**	33.7 (60)	42.1 (24)	35.3 (24)	22.6 (12)	
**CKD 4**	66.3 (118)	57.9 (33)	64.7 (44)	77.4 (41)	
***Other conditions***					
**Smokers**	38.8 (69)	24.6 (14)	41.2 (28)	50.9 (27)	0.015
**Diabetes mellitus**	36.0 (64)	26.3 (15)	41.2 (28)	39.6 (21)	0.179
**Hypertension**	95.5 (170)	96.5 (55)	92.6 (63)	98.1 (52)	0.392
***Etiology of CKD***					0.011
**Diabetic nephropathy**	18.0 (32)	14 (8)	19.1 (13)	20.8 (11)	
**Nephrosclerosis**	32.6 (58)	19.3 (11)	38.2 (26)	39.6 (21)	
**Chronic pyelonephritis**	18.0 (32)	17.5 (10)	25 (17)	9.4 (5)	
**Unknown**	15.2 (27)	22.8 (13)	10.3 (7)	13.2 (7)	
**Other**	16.3 (29)	26.3 (15)	7.4 (5)	17 (9)	
***History of CD***	38.8 (69)	17.5 (10)	42.6 (29)	56.6 (30)	0.001
**Cardiac ischemia**	11.2 (20)	5.3 (3)	13.2 (9)	15.1 (8)	0.212
**CHF**	23.0 (41)	7 (4)	26.5 (18)	35.8 (19)	0.001
**Stroke**	9.1 (16)	5.4 (3)	10.3 (7)	11.5 (6)	0.513
**Peripheral vascular disease**	16.9 (30)	12.3 (7)	17.6 (12)	20.8 (11)	0.482
**ABI**	0.92 ± 0.25	0.95 ± 0.2	0.91 ± 0.20	0.91 ± 0.33	0.552
**ABI <0.9**	37.6 (67)	28.1 (16)	42.6 (29)	41.5 (22)	0.193
***Carotid ultrasound***					
**IMT**	0.83 ± 0.17	0.72 ± 0.14	0.86 ± 0.15	0.91 ± 0.18	0.001
**Carotid plaque**	73.6 (131)	45.6 (26)	82.4 (56)	92.5 (4950)	0.001
***Atherosclerosis score***					0.001
**AS0/AS1**	25.8 (46)	54.4 (31)	16.2 (11)	7.5 (4)	
**AS2**	53.9 (96)	33.3 (19)	58.8 (40)	69.8 (37)	
**AS3**	20.2 (36)	12.3 (7)	25 (17)	22.6 (12)	
***Laboratory data***					
**Glucose (mg/dl)**	112.7 ± 39.1	107 ± 32	118 ± 45	111 ± 37	0.283
**Cholesterol (mg/dl**	166.2 ± 30.9	171 ± 31	161 ± 32	168 ± 29	0.164
**Albumin (g/dl)**	4.3 ± 0.3	4.3 ± 0.4	4.3 ± 0.2	4.3 ± 0.2	0.629
**CRP (mg/L)**	5.2 ± 6.7	4.7 ± 5.3	4.8 ± 4.8	6.1 ± 9.7	0.468
**Serum creatinine (mg/dl)**	2.5 ± 0.7	2.5 ± 0.8	2.5 ± 0.7	2.6 ± 0.7	0.731
**eGFR (ml/min)**	27.2 ± 9.6	27.5 ± 9.9	28.2 ± 10.4	25.5 ± 7.9	0.309
**Calcium (mg/dl)**	9.2 ± 0.47	9.3 ± 0.46	9.2 ± 0.51	9.2 ± 0.41	0.311
**Phosphate (mg/dl)**	3.8 ± 0.6	3.9 ± 0.8	3.7 ± 0.5	3.8 ± 0.6	0.090
**CaxP product (mg**^**2**^**/dl**^**2**^**)**	34.8 ± 6.0	36.4 ± 7.3	33.6 ± 4.7	34.6 ± 5.5	0.036
**Bone ALP (ug/L)**	18.5 ± 9.7	17.4 ± 6.2	18.2 ± 9.0	20.1 ± 12.9	0.336
**PTH(i) (pmol/L)**	15.6 ± 9.9	16.2 ± 10.1	14.8 ± 9.9	15.9 ± 9.9	0.711
**25(OH)vitamin D (ng/ml)**	22.6 ± 13.6	23.5 ± 17.6	22.6 ± 12.7	21.5 ± 9.1	0.898
**1.25(OH)**_**2 **_**vitamin D (pg/ml)**	16.5 ± 10.4	15.7 ± 8.8	17.6 ± 12.7	15.9 ± 8.4	0.509
**FEP (%)**	35.8 ± 11.0	35.6 ± 11.4	36.2 ± 10.7	35.6 ± 11.2	0.931
**FENa (%)**	2.4 ± 1.1	2.4 ± 1.1	2.3 ± 0.9	2.6 ± 1.3	0.357
**FECa (%)**	0.95 ± 0.8	0.96 ± 0.9	0.99 ± 0.9	0.89 ± 0.5	0.790
**FGF23 (RU/ml)**	153.8 ± 111.5	136.1 ± 72.1	130.8 ± 81.9	202.4 ± 156.9	0.001
**Soluble α-klotho (pg/ml)**	482.2 ± 223.1	491 ± 250	466 ± 193	493 ± 230	0.739
**Protein Intake (gr/Kg/day)**	1.01 ± 0.27	1.01 ± 0.27	1.02 ± 0.28	1.02 ± 0.28	0.977
***Medication***					
**Calcium-containing P binders**	12.9 (23)	19.3 (11)	8.8 (6)	11.3 (6)	0.202
**Oral vitamin D3**	39.3 (70)	36.8 (21)	45.6 (31)	34 (18)	0.386
**Oral active vitamin D**	4.5 (8)	5.3 (3)	5.9 (4)	1.9 (1)	0.659
**Antiplatelet**	48.3 (86)	31.6 (18)	50 (34)	64.2 (34)	0.002
**Statins**	70.8 (126)	59.6 (34)	76.5 (52)	75.5 (40)	0.080
**Diuretics**	64.6 (115)	56.1 (32)	60.3 (41)	79.2 (42)	0.025
**Loop diuretics**	77.3 (85)	67.7 (21)	79.5 (31)	82.5 (33)	0.311

Data are presented as % *(n)* = Percentage (number of patients) or Mean ± SD; P values were determined by Chi squared tests (or Fisher exact test in case of expected frequencies lower than 5) for comparison of frequencies, and Kruskal Wallis tests for quantitative variables. Abbreviations: *KI*, Kauppila Index; *BMI*, Body mass index; *BP*, Blood pressure; *CD*, Cardiovascular disease; *CHF,* Cardiac heart failure; *ABI*, Ankle, brachial index; *IMT*, Intima-media thickness; *CRP*, C reactive protein; *eGFR*, Estimated GFR; *Bone Alp*, Bone alkaline phosphatase; *FEP, FECa* and *FENa* indicate fractional excretion of phosphate, calcium and sodium, respectively; *AS*, Atherosclerosis score.

In bivariate analysis, KI associated positively with age (r: 0.419; p < 0.001), pulse pressure (r: 0.209; p = 0.005); IMT (r: 0.320; p < 0.0001) and log FGF23 (r: 0.242; p = 0.001). Also, KI was higher at later stages of CKD (3 vs. 4: mean (SD) of 2.8 (3.29) vs. 4.5 (4.63); Mann–Whitney p = 0.023), in patients with carotid plaques (4.9 (4.47) vs. 1.3 (2.25); p < 0.001; smokers (4.8 (4.05) vs. 3.4 (4.37); p =0.004), history of cardiovascular disease (5.3 (4.53) vs. 3.0 (3.90); p < 0.001) or receiving diuretics (4.5 (4.33) vs. 2.8 (4.03); p = 0.005). Interestingly, serum PTH, 25(OH)D, or calcitriol levels did not associate with KI.

Multivariate regression analyses, using KI as a quantitative variable (Table 2), identified which of the variables listed in Table 1 were independently associated to AAC in all patients (Model 1), or in patients with an estimated GFR below 30 ml/min (Model 2). The latter analysis was conducted to disregard effects on VC that could result from marked differences in renal function. Table 2 shows that age, male gender, CKD stage, presence of carotid plaques and the log of serum FGF23 associated positively with KI values, but FEP associated negatively. LR tests confirmed that neither markers of abnormal mineral metabolism (high serum P or PTH, or low vitamin D metabolites) nor any of the other variables tested or conditions predisposing to VC in individuals with normal renal function (smoking, diabetes, congestive heart failure) were independently associated with KI scores.

**Table 2:** Multivariate linear regression analysis of factors associated with the severity of abdominal aortic calcification

	**Model 1**			**Model 2**		
**Variable**	**B**	**95% CI**	**p**	**B**	**95% CI**	**p**
Intercept	−11.568	−17.218, 5.917	<0.001	−10.887	−19.151; -2.622	0.010
Male sex	1.237	0.058; 2.417	0.040	1.490	−0.075; 3.055	0.062
Age (y)	0.108	0.055; 0.162	<0.001	0.097	0.025; 0.168	0.009
CKD Stage 4 vs. 3	1.704	0.349; 3.058	0.014	N/A	N/A	N/A
Carotid plaque	1.978	0.560; 3.396	0.007	2.491	0.429; 4.552	0.018
FEP	−0.071	−0.127; -0.015	0.013	−0.083	−0.154; -0.012	0.023
Ln(FGF23)	1.488	0.448; 2.529	0.005	1.839	0.438; 3.240	0.011

Coefficient *(B)* and 95% confidence intervals *(CI)* for variables with a statistically significant (p < 0.05) contribution to explain the magnitude of abdominal aortic calcification *(AAC)*, measured by Kauppila Index *(KI)*, in a multivariate linear regression model including all the patients (Model 1) or patients with an estimated eGFR below 30 ml/min (Model 2). The coefficients of determination (r^2^) for Model 1 and 2 were 0.301 and 0.286, respectively.

**Figure 1:**
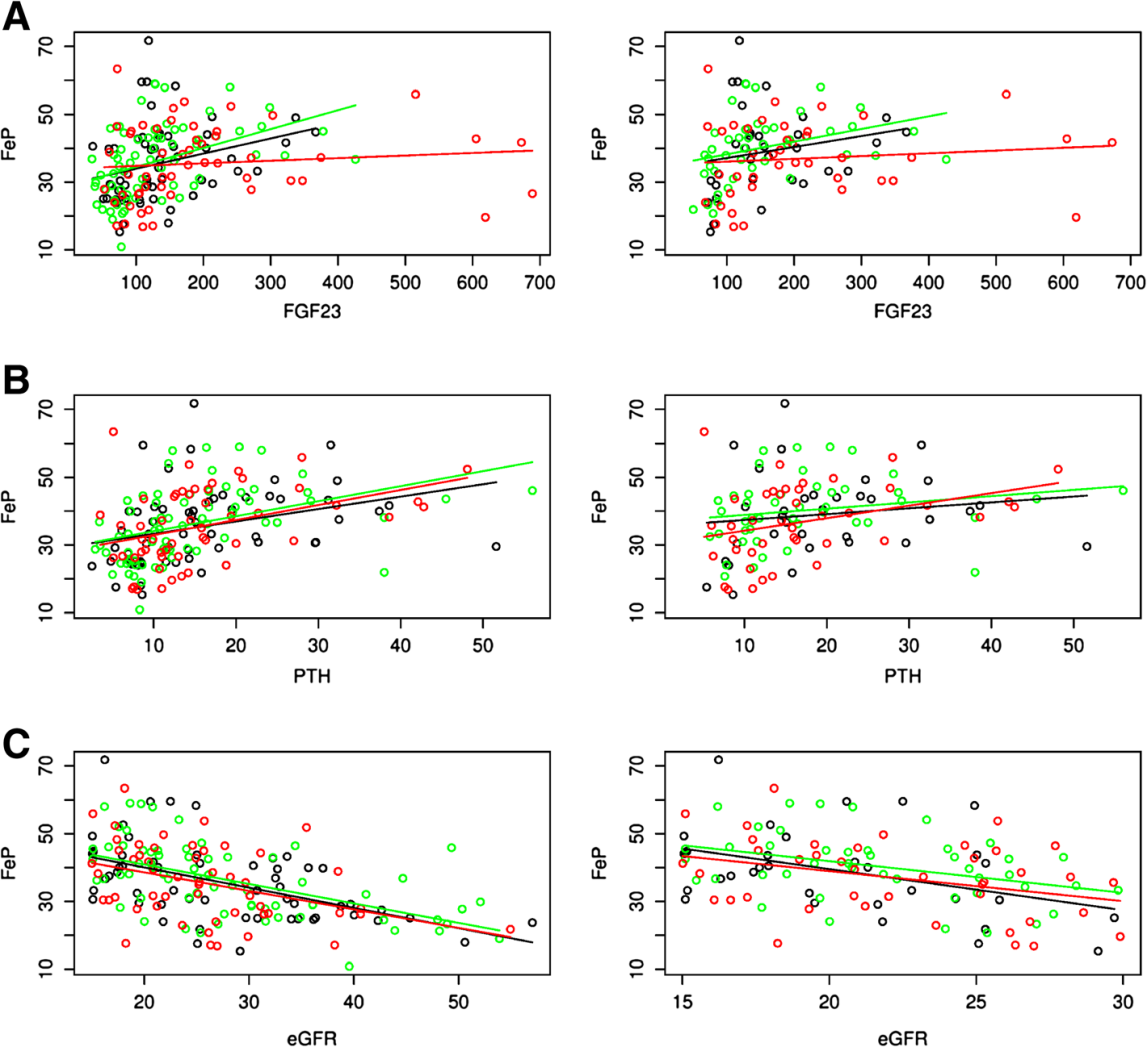
**Resistance to FGF23-driven phosphaturia in CKD patients with severe abdominal aortic calcification.** Linear regression models for the relationship between FEP and serum FGF23 **(A)**, or serum PTH **(B)** or estimated GFR **(C)** in the three subgroups of CKD patients defined by their Kauppila Index (KI; Black: KI = 0, no calcification; Green: KI = 1 to 5, moderate calcification; Red: KI > 5, severe calcification) in the whole patient population (Left side panels) or in patients with estimated GFR under 30 ml/min (Right side panels).

Because serum FGF23 and FEP increase in parallel with the progressive reductions in renal function in the course of CKD, the observed inverse association between FEP and KI suggested a role for resistance to FGF23 phosphaturic actions in the severity of AAC. Indeed, linear regression analyses between serum FGF23 and FEP as the dependent variable (Figure 1A), discriminating non-calcified patients (KI = 0) from those moderately (KI = 0-5) or severely (KI > 5) calcified, showed almost identical slopes for non-calcified (r = 0.28; p < 0.033) or moderately calcified patients (r = 0.43; p < 0.001). However, there was no correlation between FEP and serum FGF23 (r = 0.11; p = 0.44) in severely calcified patients, which supports that loss of FGF23 phosphaturic response is associated with severe AAC (KI > 5). Furthermore, high serum PTH and low GFR adversely affect VC and both are known contributors to high FEP in CKD patients. However, Figure 1B and C (left panels) show that there were no differences among KI groups either in the slopes for changes in FEP with increasing PTH (KI = 0, r = 0.32, p < 0.014; KI = 1-5, r = 0.41, p < 0.001; KI = 5, r = 0.39, p = 0.003) or with decreasing eGFR (KI = 0, r = −0.52, p < 0.001; KI = 1-5, r = −0.56, p < 0.001; KI = 5, r = −0.39, p = 0.004) in all patients. The right panels depict similar results also in patients with GFR under 30 ml/min. LR tests showed no interactions of KI tertiles on the relationship of FEP with the variables PTH or eGFR, and corroborated that only severe AAC (KI > 5) had a significant interaction (LR test; p = 0.019) on the association between FEP and FGF23 exclusively. Table 3 shows the expected increases in AAC, FEP and FGF23 in CKD stage 4 compared to stage 3.

**Table 3:** AAC and renal response to FGF23 in the course of CKD

	**CKD3 (n = 60)**	**CKD4 (n = 118)**	**p value**
AAC (KI)	2.8 ± 3.3	4.5 ± 4.6	0.02
FEP (%)	29.9 ± 8.1	38.8 ± 11.1	0.000001
FGF23 (RU/ml)	105.5 ± 87.8	178.4 ± 114.4	0.000001
Log_2_ (FEP/FGF23)	−1.64 ± 0.84	- 2.04 ± 0.78	0.002
Klotho (pg/ml)	458.6 ± 236.5	494.2 ± 216.0	0.238

**Figure 2:**
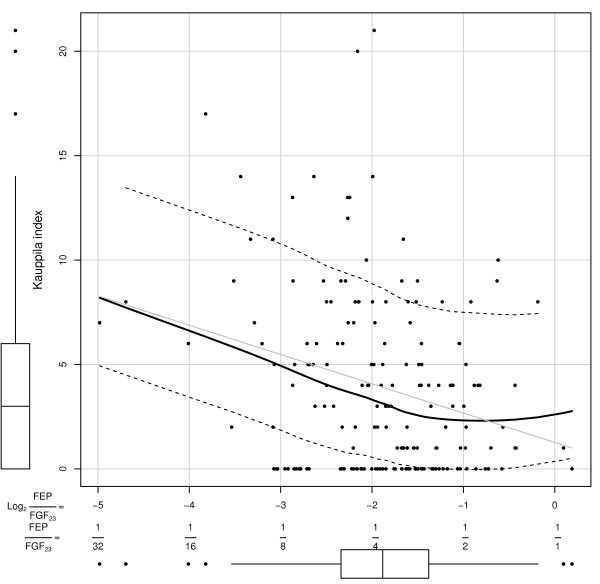
**Biphasic relationship between the severity of abdominal aortic calcification with the phosphaturic response to FGF23.** Linear regression (Gray line) and non parametric regression (Black, continuous lines) depicting the relationships between the ratio of FEP/FGF23 (in logarithmic scale (base 2): log_2_(FEP/FGF23)), in the X axis, with the Kauppila Index (Y axis). The softer continuous lanes mark the dispersion from the mean fit (see Methods for specifics). The box plot below the X axis shows that the cut-off point FEP/FGF23 = 1/3.9 coincides with the median for the log_2_(FEP/FGF23) in our patient population. Note that the cut-off point FEP/FGF23 = 1/3.9 also marks an inflexion point for quite distinct average changes for KI with changes in FEP/FGF23 ratios above and below the cut-off: No change in KI for FEP/FGF23 ratios above the cut off; Parallel increases in KI with decreases in FEP/FGF23 ratios below the cut-off. Note in the X axis, the FEP/FGF23 ratios corresponding to the values of log_2_(FEP/FGF23). The Box plot for KI distribution in our patients is depicted to the left of the Y axes.

**Figure 3:**
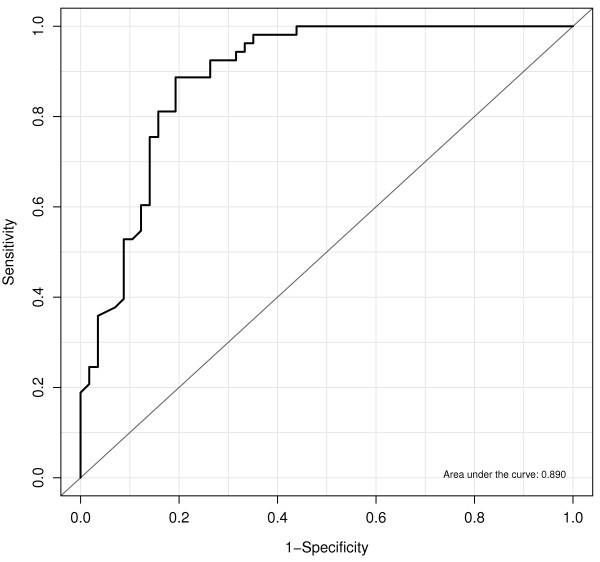
**Validation of the logistic model identifying factors independently associated with severe abdominal aortic calcification.** ROC curve depicting the high sensitivity of the logistic regression analysis of factors independently associated with severe abdominal aortic calcification (KI < 5) vs. no calcification (KI = 0) including all patients. The ROC curve includes male gender, age, CKD stage, the presence of carotid plaques, and the log_2_ (FEP/FGF23). The ratio FEP/FGF23 was introduced as a binary variable with a cutoff point of 1/3.9.

**Figure 4:**
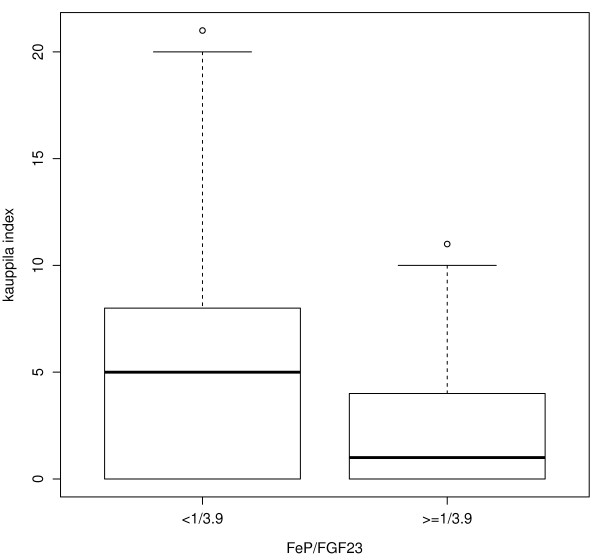
**The severity of AAC is higher in patients with a FEP/FGF23 below the threshold FEP/FGF23 = 1/3.9.** Box-plot analysis of the AAC measured by the Kauppila index (KI) in patients with a ratio FEP/FGF23 below 1/3.9 or equal or above 1/3.9. Patients were divided in two groups defined by the median of the ratio (1/3.9).

Next, in an attempt to identify a threshold for the degree of impairment in the phosphaturic response to FGF23 that associated with severe AAC, linear and non parametric plots examined the relationship between KI scores and FEP/FGF23 ratios. A logarithmic (base 2) transformation log_2_(FEP/FGF23) normalized the distribution of FEP/FGF23 ratios. Figure 2 shows a biphasic relationship between KI and the progressive reductions in the phosphaturic response to FGF23, as FEP/FGF23 ratios decrease from 1/1 to 1/32. Average KI values remained unchanged in the range of FEP/FGF23 ratios above 1/3.9 despite reductions in FEP/FGF23 ratios from 1/1 to 1/3.9. Instead, KI scores markedly increased in parallel with further reductions in FEP/FGF23 below the threshold 1/3.9. More importantly, Table 4 shows the results of logistic regression analyses conducted to identify factors that independently associate with severe AAC (KI > 5), which corroborated the significant independent association of age, CKD stage, male gender, and the presence of CP with severe AAC (KI > 5), and also with the log_2_(FEP/FGF23) < log_2_(1/3.9). Indeed, the odd ratios in these highly sensitive logistic models (Table 4 and the ROC curve in Figure 3) suggested that the presence of carotid plaques enhanced the chances of a patient to have a KI > 5 by 5 to 9-fold (Compare Model 1 and 2 in Table 3), and also, that in patients with FEP/FGF23 < 1/3.9 (serum FGF23 at least 3.9-fold higher than their respective FEP), the chances to have KI > 5 increased by a factor of 3. Accordingly, Figure 4 shows that patients with FEP/FGF23 below 1/3.9, that is, with a worse phosphaturic response to FGF23, showed a much higher average KI than patients with a FEP/FGF23 ratio over 1/3.9.

**Table 4:** Logistic regression analysis of factors associated with severe abdominal aortic calcification

**Model 1**				**Model 2**		
**Variable**	**Exp(B)**	**95% CI**	**p**	**Exp(B)**	**95% CI**	**p**
Intercept	0.003	0.000; 0.022	<.001	0.005	0.000; 0.050	<.001
Male sex	4.218	1.403; 14.207	0.014	4.167	1.050; 20.178	0.05
Age (y) - 50	1.111	1.047; 1.194	0.002	1.083	1.011; 1.177	0.037
CKD Stage 4 vs. 3	3.290	1.068; 10.773	0.041	N/A	N/A	N/A
Carotid plaque	6.131	1.605; 27.983	0.011	17.387	2.750;175.88	0.006
Cutoff point						
((FEP/FGF23) < 1/3.9)	3.915	1.346; 12.364	0.015	6.873	1.703; 35.999	0.011

Exp (B) for the intercept measures the estimated odds of KI > 5 for the reference or zero values of the explanatory variables in the model. Exp (B) of the predictor measures the odds ratio (B) associated to the variable category or 1 unit change depending of the nature of the variable. The table provides with Exp (B) and 95% confidence intervals *(CI)* for variables with a statistically significant (p < 0.05) contribution to explain the magnitude of abdominal aortic calcification *(AAC)* in a multivariate logistic regression model comparing KI > 5 vs. KI = 0 for all patients (Model 1), or among patients with an estimated GFR below 30 ml/min (Model 2). The ratio FEP/FGF23 was introduced as a binary variable with a cutoff point of (FEP/FGF23) < (1/3.9) (or equivalently, log_2_(FEP/FGF23) < log_2_(1/3.9)). The ROC curve in Figure 2 shows the high sensitivity and specificity of the logistic regression analysis in Model 1 (Area under the ROC curve = 0.89 and good model calibration, as measured by Hosmer-Lemenshow goodness-of-fit test p = 0.95). For Model 2, the area under the ROC curve = 0.899 and HL test p = 0.55.

Renal resistance to the phosphaturic actions of FGF23 develops with the progressive reductions in renal klotho in the course of CKD [29]. However, serum klotho levels were similar regardless of KI tertiles (Kruskal test; p = 0.86) or CKD stage (Table 3) suggesting that serum soluble klotho may not be an adequate marker of the magnitude of renal klotho loss in CKD.

For further validation of the independent association of a FEP/FGF23 ratio below 1/3.9 with severe AAC we used a zero-inflated model, as 32% of our patients showed a KI = 0. Table 5 shows that only log_2_ (FEP/FGF23) < log_2_ (1/3.9) was associated directly and significantly with higher KI scores in both models. Instead, age, CKD stage, male gender and carotid plaques, but not log_2_ (FEP/FGF23) < log_2_ (1/3.9) associated negatively with KI = 0. These results suggest that the progressive deterioration of the capacity of the kidney to respond to FGF23 increasing FEP may only associate with the severity of already ongoing AAC.

**Table 5:** Zero-inflated regression analysis of factors associated with abdominal aortic calcification, taking Negative binomial distribution to model KI scores

**Model 1**				**Model 2**		
**Variable**	**Exp(B)**	**95% CI**	**p**	**Exp(B)**	**95% CI**	**p**
**KI score**				**KI score**		
Intercept	1.568	0.897: 2.740	0.114	2.023	1.090; 3.756	0.026
Male sex	1.086	0.837; 1.410	0.534	1.115	0.830; 1.498	0.471
Age (y) - 50	1.022	1.004; 1.040	0.015	1.010	0.989; 1.032	0.349
CKD Stage 4 vs. 3	1.232	0.941; 1.612	0.128	N/A	N/A	N/A
Carotid plaque	1.266	0.844; 1.897	0.254	1.577	0.943; 2.636	0.082
Cutoff point						
((FEP/FGF23) < 1/3.9)	1.817	1.422; 2.320	<0.001	1.847	1.374; 2.482	<0.001
**Inflation in KI = 0**				**Inflation in KI = 0**		
Intercept	17.629	4.113; 75.551	<0.001	6.329	1.506; 26.596	0.012
Male sex	0.250	0.092; 0.682	0.007	0.305	0.078; 1.189	0.087
Age (y) - 50	0.919	0.875; 0.965	0.001	0.905	0.846; 0.969	0.004
CKD Stage 4 vs. 3	0.347	0.128; 0.942	0.038	N/A	N/A	N/A
Carotid plaque	0.271	0.100; 0.738	0.011	0.337	0.080; 1.410	0.136
Cutoff point						
((FEP/FGF23) < 1/3.9)	1.047	0.410; 2.677	0.923	0.958	0.273; 3.356	0.946

Exp (B) for the first and second intercepts are the estimated KI score and the estimated odds of KI = 0 respectively for a patient with the reference categories or zero values of the explanatory variables in the model. Additionally Exp (B) of the predictor measures the odds ratio (B) associated to the variable category or 1 unit change depending of the nature of the variable. The table provides with Exp (B) and 95% confidence intervals *(CI)* for variables with a statistically significant (p < 0.05) contribution to explain the score of abdominal aortic calcification *(AAC)* measured by KI and the high frequency of no ACC *(KI = 0)* simultaneously in a multivariate Zero-inflated regression model in all patients (Model 1), or among patients with an estimated GFR below 30 ml/min (Model 2). The ratio FEP/FGF23 was introduced as a binary variable with a cutoff point of (FEP/FGF23) < (1/3.9).

## Discussion

The results of this cross-sectional study enhance our current understanding on the key issue of the impact of renal resistance to FGF23 on critical outcomes in CKD, specifically, on the severity of AAC. To our knowledge, this is the first study to present evidence of the association between renal resistance to the phosphaturic actions of FGF23, but not to PTH-driven phosphaturia, and the degree of AAC in patients CKD-stages 3 and 4. Indeed, analysis of the relationship between KI scores and FEP/FGF23 ratios in these patients identified a FEP/FGF23 ratio, which marks a critical point for the impairment in the renal response to FGF23 phosphaturic actions that associates with a 3-fold enhancement of the risk of severe AAC.

Our analysis of the relationship of abnormalities in the phosphate/FGF23 axis with VC in CKD stages 3 and 4 supports a prior report [10] showing that high FGF23 but not high phosphate was independently associated with VC, and contradicts the recent report by the CRIC consortium in which neither serum FGF23 nor FEP were significantly associated to calcification of either the coronary artery or the thoracic aorta [14]. It is important to highlight that in the CRIC study, measurements of serum phosphate, FGF23 and FEP were obtained within a year prior to obtaining coronary and aortic calcification scores. In our study, a key role for an impaired phosphaturic response to FGF23 in AAC was first suggested by the finding that increases in FEP associated negatively with AAC in multivariate analysis, independently of the CKD stage.

To evaluate VC, we measured abdominal aortic calcification (AAC) with the semiquantitative but cost/effective Kauppila Index (KI). KI strongly correlates with coronary calcium scores from computer beam tomography [30] and reflects arterial stiffness better than coronary calcium scores [31-33]. Also, a KI > 5 can be considered as severe AAC because it was shown to increase by a factor of 3.7 the risk of adverse cardiovascular events in a large cohort of prevalent dialysis patients [34].

The accuracy of FEP measurements can also be questioned, as urinary phosphate excretion depends upon several factors, including not only the integrity of glomerular and tubular renal function, but also dietary phosphate intake (mainly in proteins), serum levels of the phosphaturic hormones FGF23 and PTH, and renal content of Klotho, the co-receptor required for FGF23 actions [35]. However, there were no differences in estimated GFR, serum PTH, or in protein intake, an estimation of P intake [36] when patients were categorized by their KI. Furthermore, FEP not only increases in response to FGF23, but also to PTH, or to decreases in eGFR. However, the slopes of the linear regression analysis of FEP with PTH or eGFR were similar among KI groups in the whole population, and also in patients with eGFR below 30 ml/min. This demonstrated an intact renal response to PTH phosphaturic actions and the expected increases in FEP with the worsening of renal function. Instead, FEP and FGF23 increased in parallel only in the non-calcified and moderately calcified patients. No increases in FEP occurred with major increases in serum FGF23 in severely calcified patients, supporting the negative association between FEP with AAC identified in the multivariate analysis. LR tests confirmed the results of the linear regression analysis. Only the slopes for FEP with increases in FGF23 were affected by the highest KI levels. Neither the slopes of the associations between FEP with PTH nor those of FEP with eGFR showed significant interactions with KI values. Although the phosphaturic response to PTH and FGF23 involves identical sodium-phosphate co-transporters in renal proximal tubular cells, the mechanisms of actions of these potent phosphaturic hormones are quite different. While PTH, through its receptor 1 and a cAMP mediated mechanism, modulates the endocytosis of the NaPi IIa cotransporters to prevent P reabsorption [37], FGF23 requires the co-receptor klotho to activate the FGFR to reduce renal content of NaPiII channels [29]. The progressive decreases in klotho in the course of CKD partly account for the renal resistance to FGF23. Thus, our findings demonstrating that FEP increases in parallel with the increases in serum PTH regardless of KI but not with the increases in FGF23 underscore our hypothesis of a role for renal resistance to FGF23 in the severity of AAC. Our results of unchanged soluble serum klotho with progressive increases in the resistance to FGF23 phosphaturic actions support previous reports suggesting that soluble serum klotho is not an accurate marker of renal klotho loss. It is likely that urinary klotho represents a better indicator of renal klotho loss, as demonstrated by Akimoto et al. [38] and supported by the new understanding of renal klotho cleavage and actions [29].

The multivariate analysis also has limitations: Its determination coefficients indicate that these models explain only 28% of the variability in KI. Also, KI values were 0 in a third of patients. However, highly sensitive logistic regression analysis confirmed the key role of impaired phosphaturic response to FGF23 in AAC, as in patients with FEP/FGF23 ratios below 1/3.9 the chances to develop severe AAC increased by 3 to 4-fold. Importantly, zero- inflated and binomial models have corroborated the accuracy of our logistic model in identifying variables associated with severe AAC in CKD patients, including the new threshold of FEP/FGF23 ratios below 1/3.9, which strongly associated to a higher risk for severe AAC. Indeed, KI scores did not change with progressive reductions in the renal response to FGF23 phosphaturic actions before reaching an almost 4-fold elevation in FGF23 without changes in FEP, but markedly increased in parallel with further reductions in the phosphaturic response to FGF23 as measured by FEP/FGF23 ratios below 1/3.9.

Our results are in agreement with a very recent report by Dominguez et al., demonstrating that the association between FGF23 levels and adverse cardiovascular outcomes was modified by FEP values. In models adjusted for CVD risk factors, kidney function, and PTH, those patients who had FGF23 above the median but FEP below the median had the highest risks of both all-cause mortality and CVD events [19]. In summary, the results of this cross-sectional study suggest that the evaluation of FGF23 levels in CKD patients should be accompanied by the assessment of the capacity of the damaged kidney to induce an adequate phosphaturic response. However, prospective studies are necessary to validate this cut-off of FEP/FGF23 = 1/3.9 as a predictive marker of the degree of renal resistance to FGF23 phosphaturic actions which, if surpassed, will markedly enhance the chances of severe AAC.

Also, as proven for hemodialysis patients [23], atheromatosis is a risk factor for AAC in non dialyzed CKD patients. The logistic analyses showed that in patients with carotid plaques the chances of severe AAC increased by 5 to 9-fold, while the zero-inflated model corroborated the negative association between age, male gender, CKD stage, and also of carotid plaques with the number of patients with KI = 0. Undoubtedly, proper patient management to attenuate the onset and/or progression of VC in early CKD should rightly focus on the prevention/treatment of co-morbid conditions predisposing to atheromatosis.

## Conclusions

A FEP/FGF23 > 1/3.9 may help protect CKD patients from severe AAC independently of age, gender, CKD stage and the presence of clinical atheromatosis in the carotid arteries. Undoubtedly, these findings need to be validated in prospective, well powered clinical trials.

## Competing interests

The authors declare that they have no competing interests.

## Authors’ contributions

LC obtained all the patient’s data and designed the study. AD helped in the analysis of the data and in the writing of the manuscript. FS helped in the analysis of the data. MMA performed the statistical analysis. JMV and EF designed the study, analyzed the data, wrote the manuscript and obtained funding. All authors read and approved the final manuscript.

## Authors’ information

LC and AD share first authorship.

JMV and EF share senior authorship.

## Pre-publication history

The pre-publication history for this paper can be accessed here:

http://www.biomedcentral.com/1471-2369/14/221/prepub
